# Impact of proteomics investigations on gastric cancer treatment and diagnosis 

**Published:** 2019

**Authors:** Mohammad Rostami-Nejad, Mostafa Rezaei-Tavirani, Vahid Mansouri, Zahra Akbari, Saeed Abdi

**Affiliations:** 1 *Gastroenterology and Liver Diseases Research Center, Research Institute for Gastroenterology and Liver Diseases, Shahid Beheshti University of Medical Sciences, Tehran, Iran*; 2 *Proteomics Research Center, Faculty of Paramedical Sciences, Shahid Beheshti University of Medical Sciences, Tehran, Iran*; 3 *Laser Application in Medical Sciences Research Center, Shahid Beheshti University of Medical Sciences, Tehran, Iran*

**Keywords:** Gastric cancer, Biomarker, Proteomic

## Abstract

Gastric cancer is one of the epidemics diseases with a high mortality rate in different countries. It causes many health problems in the world every year. It affects the digestive tract, and in advanced cases, its treatment has many difficulties. Early detection of cancer in different parts of the gastrointestinal tract can be accompanied by inexpensive treatment. As cancer cells make different biomarkers during different stages of the disease, researchers are looking for different biomarkers for gastrointestinal cancers detection. On the other hand, with the advent of advanced techniques such as proteomics and the discovery of a large number of proteins related to gastrointestinal cancer, finding the role of these proteins is essential. Indeed, the function of large amounts of these proteins has remained unknown.

Data from databases such as genes and proteins associated with gastrointestinal cancers were collected and the proteomic data of these databases were analyzed to find a clear perspective of the impact of proteomics in gastric cancer management.

The role of heat shock proteins, metabolic proteins, membrane binding proteins, galectins, prohibitins, S100 proteins, and many different types of proteins in gastric cancer was highlighted. This article reviewed proteomic researches in cancer-related areas of the gastric cancer in order to evaluate the findings of researchers.

## Introduction

 Gastric cancer mortality is the second prevalent cancer in the world (1, 2). Early onset gastric cancer pathogenesis is still unclear (2). Most gastric cancers occur in a sporadic manner and over the age of 45 years old in affected people (3). Approximately 5% of patients survive longer than 5 years after treatments in the patients diagnosed in the late stages of GC. Thus, there is motivation for early GC detection of non-invasive biomarkers, earlier than development of GC metastasis (4). Extensive research has been done on tissue (5), blood (6), and body fluids as well as tumor cells (7) to find gastric cancer (GC) biomarkers (6), with emphasis on finding proteins, DNA (8), and RNA (9). Early detection of GC could assist in suitable treatment of disease (10). Recently, proteomics technology as an efficient tool has been able to help researchers to identify different proteins involved in GC (11). 

There are not any statistical evidence to identify GC biomarkers and proteomics researches could assist in identifying the molecular basis of GC (12). Understanding the mechanism of GC is one of the goals which proteomics could contribute to (11). One of the methods used in the proteomics is mass spectrometry (MS), and the researchers have come up with several findings via this technique on GC. Yang J et al. used magnetic based purification and matrix-assisted laser desorption/ionization time-of-flight (MALDI-TOF) mass spectrometry in the serum of GC patents to identify SERPINA1 and ENOSF as potential GC protein biomarkers (13). Different sources and techniques were used to identify biomarkers of GC. WU W et al. represented a simple MS-based scoring for biomarkers to detect the early period of GC from gastric fluid and serum (4). They found relations between the levels of PRB2, SERPINA1, Elastase 3A, CystD, and CELA3B expression in GC progression (4) They believed that three-biomarker panel of CystD+PepA-Ela3A could be sufficient for GC diagnosis with 95.7% sensitivity. The following parts refer to the highlighted findings about the role of different types of proteins in GC onset and development via proteomics. 

## Methods

**Figure 1:**
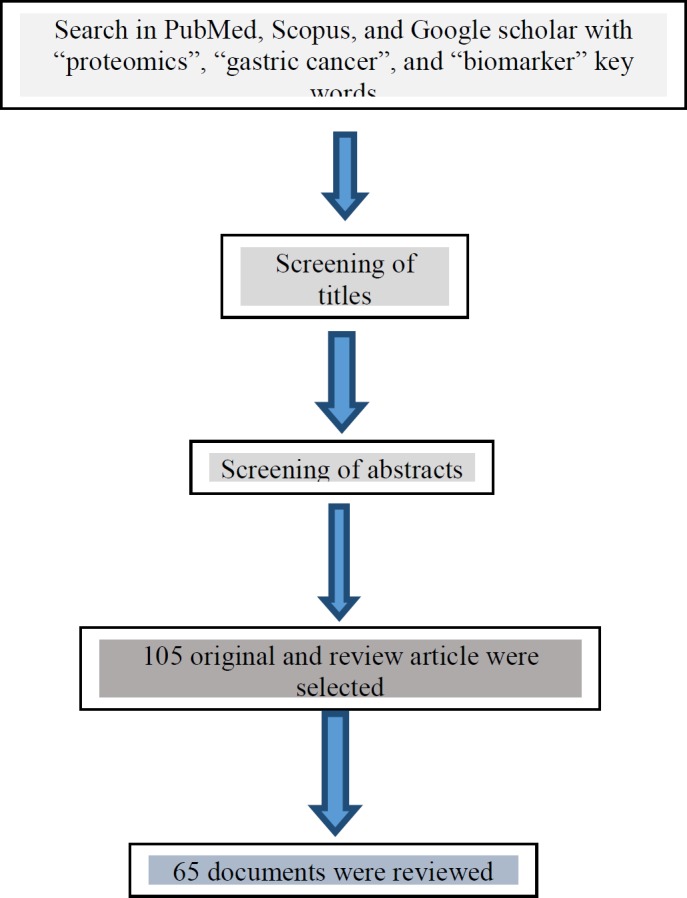
Schematic representation of the search process

Data were obtained from PubMed, Scopus, and Google scholar, from 105 articles. The search process is shown in Figure 1. Among the 105 full texts, 65 documents were selected to review. 

## Results

**Heat Shock Proteins**

 There are some heat shock proteins reported in several proteomics approaches as heat shock protein 27 (HSP 27) with overexpression in GC (14-17). Other studies revealed the upregulation of other heat shock proteins such as HSB60 (18) and HSB70 (19). In one study, the overexpression of HSP70 was observed in GC treated by administration of N-methyl-N'-nitro-N-nitrosoguanidine in rats (20). Other researchers have reported the role of HSP90 (21) and HSP105 (21, 22) in GC. HSP s are chaperons cells synthesize against stressful stimuli for survival of cells (23). However, there has been no evidence of a link between HSO70 expression and pathological characteristics (12) For example, in one study, diminished expression of HSP27 in GC cells derived from lymph nodes compared to GC cells from tumors was reported (24), while the relationship between HSP27 and the size of tumor as well as distant of metastasis was mentioned by other articles (25). The increased expression of HSP60 and HSP90 has been reported in invasive cell line compared to non-invasive cell line (21). On the other hand, decreased expression of HSP90 in GC cells derived from lymph node metastasis is inconsistent with other studies in increasing its expression (24). The drug inhibitors of HSP90 were tested clinically and it seems they could affect GC cells (26). Research has suggested that the combination of HSP inhibitors with other anticancer drugs could be beneficial in clinics to treat GC (26).

**Metabolic proteins**

Proteomic studies have revealed the overexpression of ENOA protein in GC cells compared to nonneoplastic gastric cells with or without lymph node metastasis (16,28). Capello M et al. suggested the role and function of ENOA in the metastasis process of GC and its functions (28). The diminished expression of GKN1 was reported in GC cells and ENOA protein could regulate GKN1 activity (17,29). Through the downregulation of ENOA, GC cell cycle was arrested and it was equal to overexpression of GKN1 (30). ENOA overexpression leads to tumor growth by glycolysis and pyruvate synthesis (28,31). Metabolic proteins of Krebs cycle and oxidative phosphorylation were downregulated in GC cells, according to proteomic studies (32, 33). The metabolic profile of GC cells is different to non-metastatic gastric cell profiles (15). Proteomic studies suggested Warburg effect in GC cells by forming lactate from glucose through glycolysis (34). Glucose oxidation is essential for synthesis of proteins and lipids as well as nucleic acids during cell divisions where high glycolysis is an advantage for GC tumor cells (35, 36). Forced transition of the Krebs cycle from glycolysis processcould be used as a treatment in gastric cancer (15).

**Membrane binding proteins**

Anexins, as membrane binding proteins, are calcium dependent. These intracellular proteins can form membrane bond plexus in the surface of cell membrane to interact with other proteins for different membrane functions as differentiation, migration, and dynamics of membrane (37). The increase in ANXA2 has been reported by several proteomics analyses in GC cells (32,38). ANXA2 expression increases in tumors with lymph node metastasis as compared to non-neoplastic gastric cancer cells (16). Another study revealed ANXA2 overexpression in invasive GC cells compared to non-invasive cells (39). Tumor size and location, differentiation, vessel invasion, and lymph node metastasis could affect ANXA2 overexpression (40). Its overexpression could maintain the malignancy and motility of GC cells (41). Some proteomic studies reported diminished expression of ANXA1 in GC cells (21), but other studies in contrast reported overexpression of ANXA1 (16). Nevertheless, ANXA1 overexpression leads to GS invasion as well as lymph node metastasis, and it is linked with prognostic factors such as venous and lymphatic invasions and advanced stages of GC (42, 43). However, other studies suggest ANXA1 expression during the early stages of gastric cancer (44). Proteomics investigation results have demonstrated overexpression of ANXA3, ANAXA5, and ANAXA13 in GC cells (16,45-47). Decreased expression of ANXA3 would suppress migration and invasion characteristics of GC cells (48). ANXA6, as a tumor suppressor factor of GC cells, acts through promotor mutilation (498). The overexpression of ANAXA7 in GC patients leads to reduction of survival rate vice versa (50). Reduction of ANAXA10 in GC cells and low survival rate have been reported previously. A proteomic analysis revealed the regulatory duty of ANAXA10 in GC cells proliferation (551). It is suggested that ANAXA 10 may act as a tumor suppressor in GC cells, and its expression as ANAXA7 in intestinal and diffused type of GC cells is not similar (52). These results remarkably demonstrate the prominent action of ANAXA s in GC cells development.

**Galectins roles**

Galectins (GLA) could have a role in GC development by resistance to cell death, continuing proliferative signaling and resistance to cell death as well as activation of metastasis (53). GLA1 expression in metastatic cell line is associated with the size of tumor and metastasis of lymph node as well as survival rate of GC patients (54, 55). Proteomic approaches have revealed GLA4 and GLA2 overexpression in GC cells (32, 56). GLA2 overexpression is associated with advanced stages of GC and lymph node metastasis; thus, loss of GAL2 could play an important role in GC aggression (56). GLA3 expression in GC is reduced which is associated with distant metastasis (39). Poor expression of GLA3 equals to poorer prognosis of GC and other types of cancer (57-59). Several studies are required to improve the treatment of GC with expression changes of galectins.

**S100 proteins**

S100 proteins are involved in several biological functions in cells/as proliferation and motility (60) plus chemotactic and angiogenesis activities. Several S100 proteins could bind to annexins to form a cytoskeleton and are perhaps involved in cancer cell development (61). Overexpression of S100A2 protein could reduce the ability of GC cells to invade, and upon of reduction of S100a expression, the invasive ability of GC cells increased. Down-regulation of S100A2 protein in gastric carcinoma relative to the adjacent non-cancerous gastric tissues is reported by Ying Fu liu etal. (62) 

**Prohibitions**

Proteomic approaches have revealed the overexpression of prohibitions in GC (63). However, other studies of GC showed decreased expression of prohibitions (64). Initiation of GC and tumor differentiation is a result of reduced expression of prohibitions (65).

**Table 1:** The discussed proteins which were involved in gastric cancer are presented

R	Protein	Regulation	Ref.	Additional explanation
1	HSP27	up	14-17	It is down-regulated in GC cells derived from lymph nodes compare to GC cells from tumors (23).
2	HSP60	up	18, 21	
3	HSP70	up	19-20	
4	HSP90	up	21	It is down-regulated in GC cells derived from lymph node metastasis (23).
5	HSP105	up	22	
6	ENOA	up	16, 28	
7	GKN1	down	28	
8	ANXA1	Up and down	16	Over expression is related to advanced stages of GC (41, 42)
9	ANXA2	up	32, 38	
10	ANXA3	up	16, 45-47	
11	ANXA5	up	16, 45-47	
12	ANXA6	down	49	
13	ANXA7	up	50	
14	ANXA10	down	51	
15	ANXA13	up	16, 45-47	
16	GLA1	up	54	
17	GLA2	down	55	
18	GLA3	down	39	
19	GLA4	up	32	
20	S100A2	down	62	
21	Prohibitins	up	63	Down regulation also is reported (62)
22	EPHA2	up	68	
23	CALD	down	24	
24	CAPG	up	69	
25	CRIP1	up	67	

**Different types of proteins**

Proteomic studies have identified other proteins involved in GC. Some of them are named as EPH receptor A2 (EPHA2), caldesmon (CALD), intestinal cysteine-rich protein 1 (CRIP1), and macrophage capping protein (CAPG)(66-68). Proteomic analysis has revealed CAPG overexpression in GC cells with lymph node metastasis (69). CALD expression was reduced in GC cells causing development of migration and invasion; thus it may have a critical role in progress of GC (24). The summary of findings is tabulated in the table 1.

## Conclusion

The proteins emphatically highlighted in this review were found by high throughput screening methods. They could have a major role in GC. Proteomics technique may assist in understanding the mechanisms involved in tumor phenotype. Also, increasing the gastric carcinogenesis knowledge could assist in improving the treatment methods. However, proteomics studies of GC in the elementary stages and long distance are still required to obtain the exact biomarkers involved in GC for essential useful diagnosis and treatment of disease. Meanwhile, the heterogeneity of tumors requires different several biomarkers where proteomics could assist in finding them. 

## Conflict of interests

The authors declare that they have no conflict of interest.
